# Impaired surface membrane insertion of homo- and heterodimeric human muscle chloride channels carrying amino-terminal myotonia-causing mutations

**DOI:** 10.1038/srep15382

**Published:** 2015-10-27

**Authors:** Katharina Ronstedt, Damien Sternberg, Silvia Detro-Dassen, Thomas Gramkow, Birgit Begemann, Toni Becher, Petra Kilian, Matthias Grieschat, Jan-Philipp Machtens, Günther Schmalzing, Martin Fischer, Christoph Fahlke

**Affiliations:** 1 Institut für Neurophysiologie, Medizinische Hochschule Hannover, Carl-Neuberg-Straße 1, Hannover, Germany; 2 Hôpital Pitié-Salpêtrière, 47–83 Boulevard de l’Hôpital,Paris, France; 3 Abteilung Molekulare Pharmakologie, RWTH Aachen University Templergraben 55, Aachen, Germany; 4 Institute of Complex Systems-Zelluläre Biophysik (ICS-4), Forschungszentrum Jülich, Germany

## Abstract

Mutations in the muscle chloride channel gene (*CLCN1*) cause myotonia congenita, an inherited condition characterized by muscle stiffness upon sudden forceful movement. We here studied the functional consequences of four disease-causing mutations that predict amino acid substitutions Q43R, S70L, Y137D and Q160H. Wild-type (WT) and mutant hClC-1 channels were heterologously expressed as YFP or CFP fusion protein in HEK293T cells and analyzed by whole-cell patch clamp and fluorescence recordings on individual cells. Q43R, Y137D and Q160H, but not S70L reduced macroscopic current amplitudes, but left channel gating and unitary current amplitudes unaffected. We developed a novel assay combining electrophysiological and fluorescence measurements at the single-cell level in order to measure the probability of ion channel surface membrane insertion. With the exception of S70L, all tested mutations significantly reduced the relative number of homodimeric hClC-1 channels in the surface membrane. The strongest effect was seen for Q43R that reduced the surface insertion probability by more than 99% in Q43R homodimeric channels and by 92 ± 3% in heterodimeric WT/Q43R channels compared to homodimeric WT channels. The new method offers a sensitive approach to investigate mutations that were reported to cause channelopathies, but display only minor changes in ion channel function.

Myotonia congenita is an inherited human disease characterized by muscle stiffness, muscle hypertrophy, and sustained muscle contraction upon action and percussion^1^,^2^. The delayed muscle relaxation is due to the occurrence of repetitive electrical discharges of affected fibers, the so-called myotonic runs. Hyperexcitability is caused by a greatly diminished sarcolemmal chloride conductance in affected muscle^3^. Skeletal muscle fibers are unique among excitable tissue in a large resting chloride conductance that results in short electrical length constants of the sarcolemma^4^. This feature is necessary for electrical stability of the muscle fiber since repetitive action potentials result in accumulation of K^+^ within the T-tubules of adult skeletal muscle. In the absence of the shunting sarcolemmal chloride conductance T-tubular K^+^ accumulation depolarizes the sarcolemmal membrane and triggers muscle action potentials in the absence of motoneuron activity^6^.

In recent years, various mutations in the *CLCN1* gene have been identified in autosomal dominant (Thomsen) and autosomal recessive (Becker) myotonia^7^,^8^. Functional consequences on hClC-1 channel gating or permeation have been elucidated by heterologous expression of mutant hClC-1^9^,^10^,^11^,^12^,^13^,^14^,^15^,^16^,^17^,^18^,^19^,^20^. In many cases, alteration of channel function predicted a significant reduction of the macroscopic chloride conductance that explains muscle hyperexcitability. However, there are also disease-causing mutations that leave functional properties of hClC-1 channels unaffected. Channels with unaltered gating and permeation might cause myotonia by impaired surface membrane insertion. However, this parameter has been rarely addressed, and so far no experimental approaches are available to study and quantify surface membrane insertion capability at the single cell level.

In a systematic screen of all 23 exons of the *CLCN1* gene in seven patients with recessive generalized myotonia (Becker) we found four missense mutations predicting amino acid exchanges Q43R, S70L, Y137D and Q160H. Q43 and S70 are located in the amino-terminus, Y137 in the B helix and Q160 in the C helix of hClC-1 chloride channels (Fig. 1a). Various myotonia-associated nonsense mutations that predict hClC-1 truncation in its amino-terminus are known^21^,^22^,^23^. However, only one pathogenic missense mutation in the hClC-1 amino-terminus – predicting the exchange of threonine by alanine at position 82 - has been reported^24^. Q43R and S70L are the most amino-terminal localized disease-causing missense mutations in the whole CLC gene family. We studied homo- and heterodimeric mutant channels in heterologous expression systems and found that all mutations leave channel function unaffected, but reduce surface membrane insertion. To compare the severity of the trafficking defects caused by the different mutations we developed a novel assay that uses a combination of cellular electrophysiology and fluorescence microscopy. This approach permitted accurate quantification of the percentage of homo- and heterodimeric channels embedded in the plasma membrane.

## Results

### Genetic screening of *CLCN1*

We tested seven patients with typical myotonic symptoms such as muscle stiffness, muscle hypertrophy and myalgia that improved during exercise for mutations in *CLCN1*. All tested patients carried different mutations in both alleles (Table 1). The co-existence of two *CLCN1* mutations in affected individuals as well as the occurrence of mutations in clinically unaffected father and mother supports a recessive inheritance mode of the disease-causing mutations.

In the index patient of family 1, we identified a novel c.128 A>G p.Gln43Arg (Q43R, exon 1) missense mutation in one allele, and an already known intronic c.180+3 A>T (IVS1+3 A>T) mutation in the other allele^25^. Affected members of family 2 exhibit a novel c.209 C>T p.Ser70Leu (S70L, exon 2) mutation together with the intronic c.1471+1 G>A (IVS13+1 G>A, intron 13)^26^ mutation. A third missense mutation located in B helix, c.409 T>G p.Tyr137Asp (Y137D, exon 3)^27^, was found in a heterozygous state in four other myotonia congenita index cases (families 3–6). In each index case it was associated with other heterozygous mutations: the recessive c.180+3 A>T splice mutation in family 3^25^, the recessive missense c.1488 G>T p.Arg496Ser^26^,^28^ in family 4, the presumably recessive c.2365-2 A>G splice site mutation (intron 19, acceptor site) in family 5, and the missense c.2564 G>A p.Gly855Glu (G855E, exon 22) in family 6. Gly855Glu (G855E, exon 22) was also found in compound heterozygous state in another patient in our laboratory. We assume that this mutation, which has never been described in the literature, is recessive. In the index patient of family 7, sequencing revealed the mutations c.480 G >C p.Gln160His (Q160H^29^, exon 4) and c.2680 C>T p.Arg894* (R894X, exon 23)^30^,^31^.

**Figure 1:**
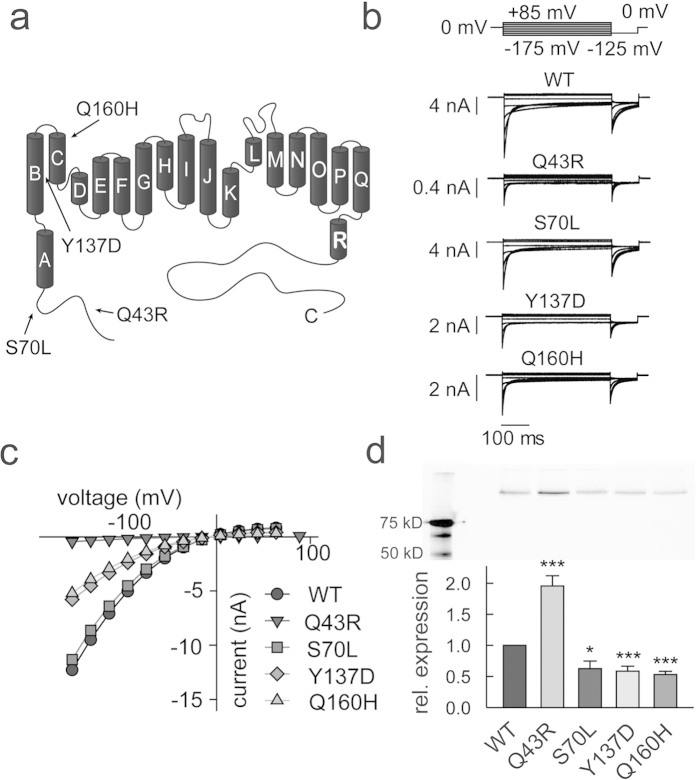
Myotonia-associated mutations modify mean current amplitudes in heterologous expression system without reducing protein expression levels. (**a**) Location of the tested mutations in a topology model of the chloride channel protein modified from Dutzler *et al.*^5^ (**b**) Representative currents of WT and mutant hClC-1 recorded under symmetric chloride conditions. (**c**) Voltage dependence of mean instantaneous current amplitudes (means ± SEM from 6–18 experiments). (**d**) Relative expression levels of WT and mutant hClC-1 channels given from relative fluorescent values (lower panel, means ± SEM from 5 experiments) and representative fluorescence scan of a SDS-PAGE gel of YFP-tagged WT and mutant hClC-1 channels in the total cleared lysate of cells that were transfected with equal amounts of plasmid DNA. The expected molecular mass of mYFP-hClC-1 is 136 kDa. In each experiment lysate samples were adjusted to equal total protein amounts before loading the gel. (Student’s two-tailed t-test with *p < 0.05 and ***p < 0.001).

The disease-associated mutations Q43R, S70L, Y137D and Q160H predict amino acid substitutions within a region of the hClC-1 subunit (Fig. 1a) whose function is insufficiently understood. We decided to investigate the functional consequences of these mutations in transfected mammalian cells.

### Functional characterization of mutant hClC-1

To obtain a homogenous population of homodimeric channels we transfected 0.5–1.0 μg pSVL-mYFP-hClC-1 encoding WT or mutant hClC-1 in HEK293T cells and studied transfected cells through whole-cell patch clamping. Figure 1b shows representative whole-cell current recordings from cells expressing WT, S70L, Y137D or Q160H hClC-1. For WT as well as for mutant channels, currents increased instantaneously upon voltage steps to negative potentials followed by a slower decrease of the current amplitude due to voltage-dependent channel gating^31^,^32^. Current responses to depolarizing voltage steps were time-independent.

When using similar transfection protocols as for WT and the other mutants, we observed currents with characteristic hClC-1 properties only in a small number of cells expressing Q43R hClC-1. These currents had very small peak current amplitudes (at −155 mV: 0.17 ± 0.04 nA, n = 6), and augmenting the amount of transfected DNA did not significantly enhance these currents. We reasoned that the number of Q43R hClC-1-YFP in the surface membrane might be increased by further enhancing the expression levels of this particular mutant protein. We therefore subcloned the coding region of Q43R hClC-1-YFP into pRcCMV, an expression plasmid with stronger promoter and transfected larger DNA amounts (2 μg) than for WT. Under these conditions, we regularly observed small currents that resembled hClC-1 in time and voltage dependence (Fig. 1b). Figure 1c gives mean current amplitudes from cells transfected with pSVL-mYFP-hClC-1 encoding WT, S70L, Y137D or Q160H hClC-1 or with pRcCMV-mYFP-Q43R hClC-1. Whereas S70L and WT currents were comparable, Y137D and Q160H reduced macroscopic current amplitudes by about 50%. Despite using a stronger promoter for heterologous expression cells transfected with pRcCMV-mYFP-Q43R hClC-1 exhibited by far the smallest current amplitudes.

To test whether the different macroscopic current amplitudes in cells expressing WT or mutant hClC-1 are due to impaired mutant protein biogenesis, we transfected equal amounts of WT and mutant pSVL-mYFP-hClC-1 in HEK293T cells and compared expression levels by SDS PAGE (Fig. 1d). Scanning the wet polyacrylamide gels with a fluorescence scanner revealed a single protein band with a molecular weight corresponding to values expected for channel fusion protein. Additional fluorescent protein fragments were absent demonstrating that proteolytic cleavage is negligible and that the number of channel proteins is proportional to YFP fluorescence amplitudes. Quantitative analysis of fluorescence intensities demonstrates slight, but significant decreases in expression levels of S70L, Y137D or Q160H hClC-1-YFP, but greatly increased levels for Q43R hClC-1-YFP. The reduction in macroscopic current amplitudes in cells expressing mutant channels is therefore not due to impaired protein biosynthesis.

**Figure 2:**
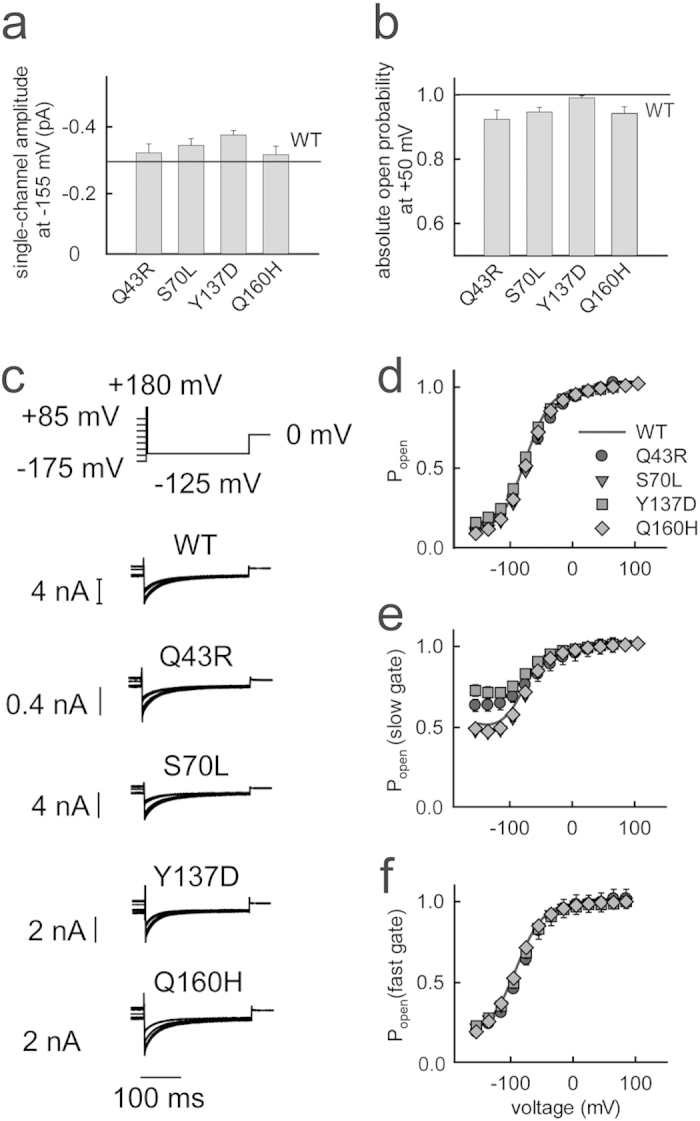
Functional properties of homodimeric mutant channels. (**a**,**b**) Mean single-channel current amplitudes at −155 mV (**a**) and absolute open probabilities at +55 mV (**b**) as obtained from non-stationary noise analysis for mutant hClC-1 (means ± SEM from 4–5 experiments). Solid lines represent WT unitary current amplitude and absolute open probability. (**c**) Representative tail currents from experiment to determine the voltage dependence of the slow common gate. (**d**) Voltage dependence of the probability of WT or mutant hClC-1 channels to be open. (**e**,**f**) Activation curves of the slow (**e**) and the fast (**f**) gate of WT or mutant hClC-1 channels (means ± SEM from 7–14 experiments).

Mutations can reduce macroscopic chloride conductances by modification of the unitary current amplitude or the absolute open probability. We employed non-stationary noise analysis to determine these two values. Supplementary Fig. S1 illustrates a representative analysis for WT hClC-1 (Supplementary information). Variance values were plotted versus mean current amplitudes (Supplementary information, Supplementary Fig. S1b), and a quadratic equation was fitted to the data, providing the number of channels in the cell surface membrane and the unitary current amplitude (N = 18,000; i = 0.28 pA at −155 mV)^33^. Fig. S1c and d (Supplementary information) display the same type of analysis for mutant Q43R channels (N = 17385; i = 0.32 pA at −155 mV). We observed unaltered single-channel current amplitudes for Q43R as well as for all the other tested mutations (Fig. 2a). Moreover, we could not detect significant alterations of the maximum absolute open probability (Fig. 2b). We next determined the voltage dependence of relative open probabilities for WT and mutant channels by plotting instantaneous current amplitudes at a voltage step to −125 mV versus the preceding potential (Fig. 2d). Since WT and mutant hClC-1 display a maximum absolute open probability of 1 at positive potentials and since none of the mutants causes significant alterations of this maximum value, such relative open probabilities are in all cases identical with absolute open probabilities^31^. For all mutant channels we observed unaltered voltage dependences of open probabilities.

hClC-1 channels are known to exhibit two different gating processes. Fast gating opens and closes individual protopores, while slow cooperative gating steps act on both protopores together^13^. Insertion of a short pulse to +180 mV into the pulse protocol prior to the test step to −125 mV (Fig. 2c) fully activates the fast gate and permits acquisition of the relative open probability of the slow gate^31^,^34^ (Fig. 2e). Assuming that fast and slow gating occur independently of each other, open probabilities of the fast gate were calculated by dividing the channel open probability by the relative slow gate activation curve (Fig. 2f). S70L and Q160H did not modify the slow gate, whereas Q43R and Y137D increased the minimum open probability of the slow gate. These slight changes left the total open probability of the channels virtually unaffected since open probabilities of the fast gate are very low at negative potentials.

We conclude that none of the studied disease-causing mutations leads to changes in hClC-1 functions that could account for the observed reduction in whole-cell current amplitudes of Q43R, Y137D and Q160H and for the reduced resting chloride conductance in affected muscle.

### Subcellular distribution of WT and mutant hClC-1

We next investigated the subcellular distribution of WT and mutant hClC-1 channels using confocal microscopy. For these experiments we used MDCK II cells, an established model to investigate trafficking of endogenous or heterologously expressed proteins^35^,^36^,^37^. We expressed fluorescent mYFP-tagged fusion proteins of hClC-1 and examined transfected cells by confocal imaging (Supplementary information, Supplementary Fig. S2). In agreement with earlier studies^38^, WT hClC-1-YFP does not exclusively insert into the surface membrane, but causes additional staining of intracellular compartments. This contrasts the predominant sarcolemmal/T-tubular localization of hClC-1 in native tissue^39^,^40^ and is most likely caused by overexpression of hClC-1 fusion proteins (our own unpublished observations). S70L hClC-1-YFP exhibited a comparable subcellular distribution as WT hClC-1-YFP (Supplementary information, Fig. S2). The remaining mutations resulted in alterations of the subcellular distribution of mYFP-hClC-1 fusion proteins. Q160H and Y137D increased intracellular staining, with prominent appearance of mutant hClC-1-mYFP in intracellular compartment. We did not attempt to clarify into which cell compartments mutant fusion proteins are mis-targeted. For Q43R hClC-1-YFP, surface staining was absent, and almost all protein was present in intracellular compartments. For WT as well as for mutant fusion proteins, no nuclear staining was observed (Supplementary information, Fig. S2). This supports the notion that fusion proteins are fully translated and proteolytic cleavage is negligible^41^. These changes in subcellular distribution provide an explanation for the reduced whole-cell current amplitudes, but do not allow quantitative assessment of altered hClC-1 surface insertion probabilities.

### Quantification of relative membrane insertion probabilities by combined fluorescence and current recordings

**Figure 3:**
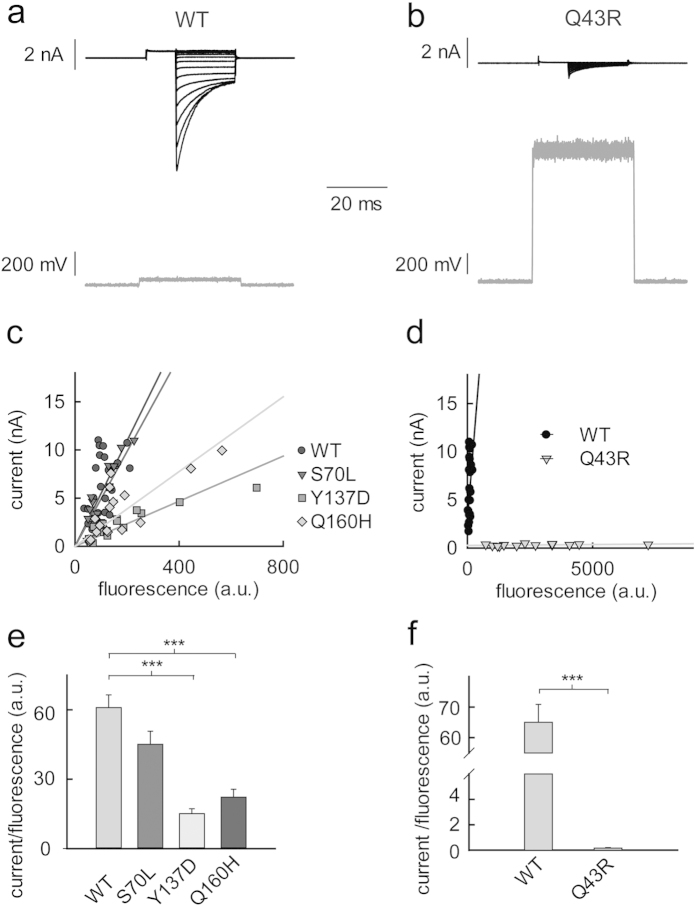
Simultaneous investigation of channel expression and surface membrane insertion. (**a**,**b**) Representative recording of the fluorescence (gray lines) and current (black lines) of cells expressing WT (**a**) or Q43R hClC-1 channels (**b**). (**c**,**d**) Correlation between instantaneous current amplitudes at −135 mV and whole-cell fluorescences for WT and mutant hClC-1. Lines represent linear regressions (regression coefficients are: 0.47 (WT) 0.77 (S70L); 0.32 (Y137D); 0.57 (Q160H); 0.35 (Q43R)). (**e**,**f**) Slopes of linear regression lines reveal relative membrane insertion rates for WT and mutant hClC-1 (means ± SEM from 13–23 experiments). Membrane insertion is significantly reduced by Q43R, Y137D and Q160H, but not by S70L (***Student’s two-tailed t-test with p < 0.001).

We developed a novel assay that combines fluorescence and current recordings to quantify changes in surface membrane insertion capability of hClC-1 by disease-causing mutations. Since unitary current amplitudes and absolute open probabilities are identical for WT and mutant hClC-1 (Fig. 2), macroscopic current amplitudes are proportional to the number of channels in the surface membrane of transfected HEK293T cells. To measure the total number of hClC-1 subunits – in intracellular organelles and the surface membrane – we expressed WT and mutant hClC-1 as mYFP fusion protein and measured the whole-cell fluorescence^42^. In these experiments we adjusted the transfected plasmid quantities so that macroscopic current amplitudes are in a comparable range (0.2–0.5 μg pSVL-mYFP-WT hClC-1, 0.5 μg pSVL-mYFP-hClC-1 encoding S70L, Y137Q, or Q160H hClC-1-YFP, or 2 μg pRcCMV-mYFP-Q43R hClC-1). Figure 3a,b show representative recordings from a HEK293T cell expressing WT hClC-1-YFP and another cell expressing Q43R hClC-1-YFP. The upper traces display current responses to a series of voltage steps consisting of an activating prepulse to +75 mV and test steps to various voltages between −175 mV and +85 mV. The lower traces depict the whole-cell fluorescence that increases upon opening of a shutter for optical excitation. Cells expressing WT hClC-1-YFP produce large current amplitudes and low whole-cell fluorescence levels, indicating a high fraction of channels inserted into the surface membrane. The use of a stronger promoter resulted in high fluorescences, but only small macroscopic current amplitudes in pRcCMV-mYFP-Q43R hClC-1-transfected cells (Fig. 3b).

Figure 3c,d display the correlation between current amplitude and whole-cell fluorescence for several cells expressing WT or mutant hClC-1-YFP. For all tested mutants we observed a linear relationship between these two variables over the tested range of fluorescence levels, indicating that channel expression did not saturate intracellular transport processes under our experimental conditions. The slopes of the linear regression lines (*I/F* = whole-cell current/whole-cell fluorescence) are proportional to the probability of channels being inserted into the surface membrane (*P*_*ins*_).

hClC-1 are double-barrelled channels which are assembled from two subunits^13^.


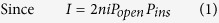



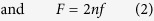



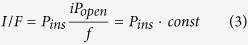


*n* is the number of all channels expressed in the cell, either in intracellular compartments or in the surface membrane, *i* the unitary current amplitude of an individual protopore, *P*_*open*_ the open probability and *f* the single-subunit fluorescence. In our analysis we assume that *f* is the same for all tested constructs. Since the YFP moiety was added to the unstructured amino-terminus of WT and mutant hClC-1, the fluorescent protein will fold independently of hClC-1, and mutations in hClC-1 are not expected to affect YFP maturation or fluorescence lifetimes or quantum yield^43^.

Comparison of regression lines provided different slopes for WT and mutant channels (Fig. 3c,e). The most pronounced effect was observed for Q43R, with a 300fold reduction of the relative insertion probability as compared to WT hClC-1 (Fig. 3d,f). No significant difference was observed for S70L (Fig. 3e). We conclude that Q43R, Y137D and Q160H impair surface membrane insertion of hClC-1.

### Q43R also reduces insertion probability of heterodimeric channels

**Figure 4:**
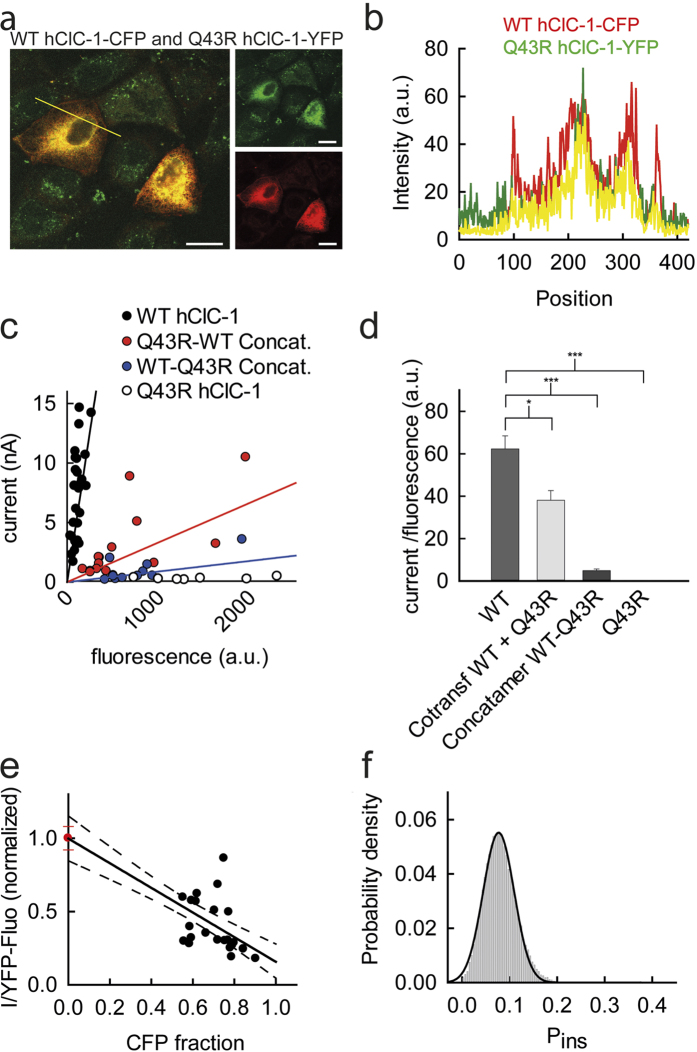
Heterodimeric channels assembled from WT and Q43R hClC-1 exhibit a reduced surface membrane insertion probability. (**a**,**b**) Confocal image (**a**) and corresponding profile intensities (**b**) for MDCK II cells co-expressing mYFP tagged WT and CFP-tagged Q43R hClC-1. Cells were co-transfected with 0.2 μg pSVL-mYFP-WT hClC-1 and 0.5 μg pSVL-mCFP-Q43R hClC-1. (**c**) Correlation between instantaneous current amplitude at −135 mV and whole-cell fluorescence for cells expressing WT or Q43R homodimers, or WT-Q43R or Q43R-WT heterodimeric concatamers. (**d**) Relative membrane insertion rates of homo- and heterodimers (means ± SEM from 11–23 experiments) (Student’s two-tailed t-test with *p < 0.05 and ***p < 0.001). (**e**) Plot of the current by YFP fluorescence ratio versus the CFP fluorescence from 21 cells co-expressing WT hClC-1-mYFP and Q43R hClC-1-CFP. I/F_YFP_ was normalized to values obtained for WT hClC-1-YFP in absence of Q43R hClC-1-CFP (fraction of CFP = 0). The y-axis intercept is determined by the mean current by fluorescence ratio from 29 cells that exclusively express WT hClC-1-YFP (shown as mean ± SEM in red). (**f**) Distribution of estimated surface membrane insertion probabilities from 50,000 bootstrap samples of the original data. Histograms have been normalized such that the integral over the range is 1 and fitted with a Gaussian function (μ=0.081, σ = 0.034).

ClC-1 channels are dimers^44^, and heterodimers are thus expected to represent a major fraction of channels in heterozygous patients. To test whether the disease-associated mutants affect also intracellular trafficking of heterodimers we focused on Q43R, the mutation with most pronounced effects on trafficking. We initially co-expressed WT and Q43R mutant channels as YFP and CFP fusion proteins, respectively, in MDCK II cells and studied the subcellular distribution of the fusion proteins by confocal imaging (Fig. 4a,b). In such cells, a large fraction of WT hClC-1-YFP fusion proteins is intracellularly retained co-localizing with Q43R hClC-1-CFP, suggesting that mutant subunits impair surface membrane insertion of WT hClC-1. However, since expression of WT hClC-1 alone also results in significant staining of intracellular compartments, this effect is difficult to quantify.

We next constructed concatameric WT-Q43R as well as Q43R-WT hClC-1 channels as mYFP fusion proteins and studied the correlation of whole-cell current and fluorescence for pure expression of heterodimers (Fig. 4c). Since concatamers contained only one fluorescent protein per protein dimer we doubled the measured fluorescence values to permit comparison with homodimeric fusion proteins. Both concatamers exhibit dramatically reduced surface insertion probabilities (Fig. 4c,d), however, they differed in the degree of insertion reduction. Whereas WT-Q43R reduced surface insertion to 2% ± 1% of WT, we observed a higher value for Q43R-WT that measured about 8% ± 1% of homodimeric WT hClC-1.

We next co-transfected WT and mutant Q43R hClC-1 DNA (0.5 μg pSVL-mYFP-WT hClC-1 and 2 μg pRcCMV-mYFP/CFP-Q43R hClC-1) and analyzed the surface membrane insertion probabilities of heterodimeric channels with a modified approach of combined fluorescence/electrophysiology. Increasing CFP-fractions upon co-expression of Q43R subunits will decrease the fraction of WT subunits (CFP-fraction + YFP-fraction = 1) and also the percentage of WT homodimers by the formation of heterodimers. Figure 4e depicts the quotient of current by the YFP fluorescence (I/F_YFP_) versus the fraction of CFP-tagged Q43R hClC-1 subunits for 25 cells. These data demonstrate that a higher percentage of CFP-Q43R hClC-1 subunits are associated with reduced I/F_YFP,_ indicating lower insertion probabilities of heterodimeric channels than of homodimeric WT channels.

Under the assumption of a binomial distribution of WT and mutant homodimeric and WT-mutant heterodimeric channels, anion currents of cells co-expressing YFP-tagged WT and CFP-tagged Q43R subunits equal to


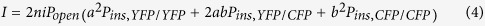


with n being the total number of channels, and a and b the fraction of WT hClC-1-YFP (a) and Q43R hClC-1-CFP (b) subunits. P_*ins*_ gives the membrane insertion probability, i the unitary current amplitude per protopore and P_open_ the absolute open probability. Using a relative insertion probability of 1 for homodimeric WT and of 0 for homodimeric mutant channels the equation simplifies to


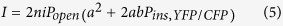


The whole-cell YFP fluorescence (F_YFP_) depends on the number of WT hClC-1-YFP subunits (2n for n channels), the fraction of YFP (a) and the fluorescence amplitude of a single YFP (f)


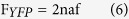


so that


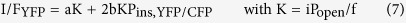


Since a + b = 1:


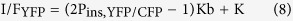


Fitting equation (8) to the plot of I/F_YFP_ versus the CFP-fraction (b) allows determination of the insertion probability of heterodimers. We determined the fit reliability and the regression error using bootstrap sampling (using 50.000 bootstrap samples with replacement as described in^45^). The thus obtained fit parameters indicate that heterodimeric channels assembled from one WT and one Q43R hClC-1 subunit exhibit a significantly decreased surface insertion probability that equals 8 ± 3% of WT homodimers (Fig. 4f).

### Q43R does not interfere with homo- or heterodimerization

**Figure 5:**
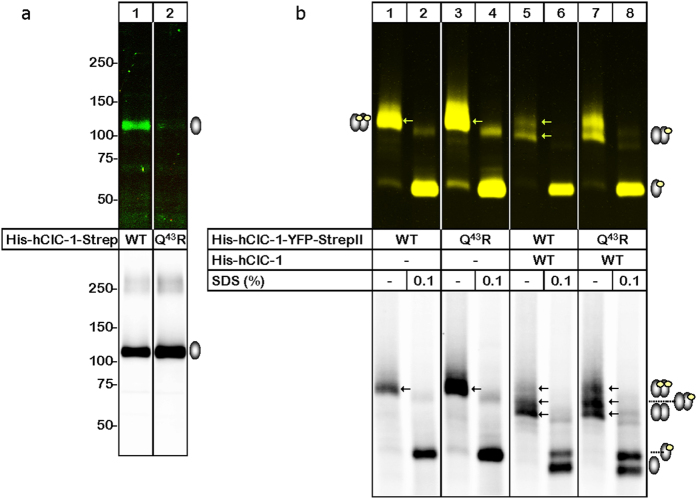
hClC-1 oligomerization is not impaired by Q43R. (**a**) SDS-PAGE gel (4–10% gradient) of WT and Q43R hClC-1 after expression in *Xenopus* oocytes and purification via StrepTactin chromatography. Proteins were visualized by scanning of plasma membrane-bound fluorescence (upper panel) or ^35^S-labeled total protein (lower panel). (**b**) Oligomeric state of WT and mutant hClC-1 channels. Indicated hClC-1 proteins were purified via metal affinity chromatography, resolved by BN-PAGE (4–12% gradient) and visualized by scanning of YFP fluorescence (upper panel) and ^35^S-labeled total protein (lower panel). The protein migration is shown both under native conditions and following partial denaturation after a 1-h incubation with 0.1% SDS at 37 °C, as indicated. The gray ovals without and with yellow balls schematically illustrate YFP-less and YFP-fused hClC-1, respectively, to indicate the native dimeric and denatured protomeric states of the corresponding protein bands. The data shown represent samples run on the same SDS-PAGE gel or BN-PAGE gel; irrelevant lanes were cropped from the figure as indicated by vertical lines. The respective source images of the individual gels are shown in Supplemental Fig. S3a and b.

To test whether WT subunits associate equally well with WT and with Q43R subunits, we expressed hClC-1 proteins either as His-hClC-1-YFP or as His-hClC-1-StrepII in *Xenopus* oocytes and purified them by metal affinity or StrepTactin chromatography. Cell surface-specific labelling of hClC-1 using a membrane-impermeant Cy5-NHS ester revealed greatly reduced incorporation of Q43R hClC-1 also into the oocyte plasma membrane (Fig. 5a, upper panel, lanes 1 and 2). This result is not attributable to a weak expression, as the [^35^S]methionine-labeled forms of the WT and Q43R hClC-1 could be purified in similar amounts from the oocytes (Fig. 5a, lower panel). Both WT and Q43R hClC-1 migrated at approximately 114 kDa in the SDS-PAGE gel, close to the calculated 110 kDa of the hClC-1 apoprotein.

We next compared the assembly efficacy of Q43R hClC-1 by BN-PAGE under conditions known to preserve the homodimeric state of hClC-1^46^. After purification WT and Q43R His-hClC-1-YFP migrated to the same position in the BN-PAGE gel in their non-denatured state, as detected by scanning the fused YFP fluorescence or the ^35^S radioactivity originating from metabolic [^35^S] methionine incorporation (Fig. 5b, lanes 1 and 3, upper and lower panels). Denaturation with 0.1% SDS resulted in complete disassembly of WT and Q43R hClC-1 homodimers (Fig. 5b, lanes 2 and 4). To assess whether the WT and Q43R hClC-1 assemble equally well with each other, we took advantage of the larger mass and the YFP fluorescence of the hClC-1-YFP fusion proteins as compared to His-hClC-1-StrepII. Co-expression of WT His-hClC-1-StrepII with WT His-ClC-1-YFP resulted in the appearance of two distinct YFP fluorescent bands in the BN-PAGE, the hClC-l-YFP homodimer and the 29 kDa smaller hClC-1-YFP/hClC-1 heterodimer (Fig. 5b, lane 5, upper panel). Exactly the same pattern of bands was seen upon co-expression of the WT His-ClC-1 with Q43R His-hClC-1-YFP (Fig. 5b, lane 7, upper panel). These data indicate that the WT hClC-1 assembles equally well with the WT and Q43R hClC-1-YFP. Upon denaturation with SDS, only one faster migrating band could be seen, the His-hClC-1-YFP protomer (Fig. 5b, lanes 6 and 8, upper panel). The ^35^S-phosphorimager scan of the BN-PAGE gel additionally made visible the YFP-less proteins, the WT and Q43R hClC-1 protomers and the WT and Q43R hClC-1 homodimers (Fig. 5b, lanes 5–8, lower panel). We conclude that Q43R does not affect the assembly capacity of the hClC-1.

## Discussion

Disease-causing mutations often result in significant alterations of ion channel gating and permeation. However, there are also many examples that leave these channel properties unaffected and merely result in changes of macroscopic current amplitudes. In these cases it is often assumed that the mutation affects subcellular trafficking of mutant ion channels. We here report a novel approach that permits quantification of surface membrane insertion of mutant muscle chloride channels at the single cell level.

We focused on two novel (Q43R and S70L) and two already known (Y137D and Q160H)^27^,^29^ myotonia-associated *CLCN1* mutations. These *CLCN1* mutations were found in seven patients who exhibited typical myotonic symptoms such as muscle stiffness that disappeared upon muscle activity (warm-up phenomenon), muscle hypertrophy and myalgias in some of the cases and typical myotonic bursts in EMG recordings (Table 1). In all cases, father and mother were asymptomatic, in agreement with a recessive inheritance mode. All patients were compound heterozygous, with one missense mutation predicting amino acid exchanges in the amino-terminus. Most myotonia-causing mutations are very rare, and compound heterozygosity is therefore a frequent finding in patients with recessive generalized myotonia congenita^12^,^14^,^17^,^25^,^26^,^29^,^33^. In these patients, three types of hClC-1 are expressed, homodimeric hClC-1 carrying one of the two mutations as well as heterodimeric channels assembled of one subunit with one mutation and a second subunit carrying the other mutation. The asymptomatic parents are heterozygous for each of the two mutations, and will thus express homodimeric mutant and WT channels together with heterodimeric channels consisting of one mutant and one WT subunit.

Q43R and S70L predict amino acid exchanges amino-terminal to the A helix, Y137D in the B helix and Q160H in the C helix of the human muscle chloride channel hClC-1 (Fig. 1a). For all tested mutations, we observed identical unitary current amplitudes and absolute open probabilities as well as virtually unchanged current kinetics (Fig. 2, Supplementary Fig. S1). However, three of the four mutations reduced macroscopic current amplitudes (Fig. 1). Since the function of mutant channels was unaltered the current reduction must have been exclusively caused by a decreased number of channels in the surface membrane. To quantify disease-associated impairment of surface membrane insertion, we simultaneously measured anion currents and fluorescences of YFP-tagged channels in individual cells. We reasoned that measuring current amplitudes is the most accurate method to determine the number of anion channels in the surface membrane. Expression of fusion proteins of hClC-1 channels and fluorescent proteins permits determination of the number of channels in intracellular compartments as well as in the surface membrane using fluorescence measurements^42^,^43^. The ratio of macroscopic current amplitude by whole-cell fluorescence provides a value that is proportional to the membrane surface insertion probability and permits comparison of WT and mutant channels in their ability to reach the plasma membrane in a single cell assay (Fig. 3).

A frequently used method to estimate the protein fraction in the surface membrane is surface biotinylation^20^. It relies on covalent reaction of biotin with the channel protein and successive non-covalent binding to neutravidin beads. Both reactions have incomplete efficiency and thus underestimate the number of surface membrane inserted as well as the number of intracellular hClC-1. Moreover, biochemical approaches require large numbers of individual cells and are thus incapable to detect differences between individual cells. Thus, surface biotinylation cannot account for heterogeneous cell populations that are caused by different protein expression levels. Subcellular channel distribution might be altered upon protein overexpression in heterologous expression systems. Our approach compares surface membrane insertion of multiple cells with different expression levels and thus provides direct information about such aberrant localization in overexpressing cells. All these features make our single cell assay more accurate and more sensitive than surface membrane biotinylation.

The surface membrane insertion probability was three- to fourfold reduced by Y137D and Q160H, and Q43R decreased the current by fluorescence ratio by almost 300. This dramatic reduction required expression of much higher numbers of Q43R fusion proteins to obtain measurable current amplitudes. However, even at these increased protein levels, we observed a linear relationship between expression levels and membrane currents, indicating that none of the distinct trafficking steps was saturated. These findings also argue against toxic effects of the high Q43R hClC-1-YFP protein expression levels that may additionally disturb targeting of the channel.

All mutations under study exclusively modify intracellular trafficking of ClC-1, and one might thus expect a correlation between current amplitude reduction in heterologous expression systems and the severity of myotonic symptoms. However, patients with Q43R did not exhibit more pronounced symptoms than patients carrying other mutations. This is in agreement with earlier reports that did not find any correlation between alterations of hClC-1 channel function in heterologous expression systems and myotonic symptoms^31^,^47^.

For S70L, we observed macroscopic current amplitudes and current/fluorescence slopes that are comparable to WT hClC-1 and were therefore unable to identify any change in function that might account for sarcolemmal hyperexcitability in the affected patient. However, several lines of evidences support the notion that S70L is a recessive mutation, rather than a benign polymorphism. The mutation was never found in more than 700 control samples sequenced in our laboratory. Affected members of family 2 are compound heterozygous carrying the c. 1471+1 G >A (IVS13 +1 G >A, intron 13) in the other allele. This intronic mutation was reported before as recessive mutation in another compound heterozygous patient^26^. Thus, we have to assume that S70L modifies hClC-1 properties that cannot be studied in a heterologous expression system. One might speculate that S70L affects hClC-1 insertion into muscle specific membrane compartments. At present, it is not clear whether hClC-1 is exclusively expressed in the sarcolemma^40^, or uniformly distributed over sarcolemma and t-tubular invaginations^39^. It appears possible that S70L might either induce or prevent insertion into the t-tubulus and thus modify sarcolemmal excitability^48^. At present, we are unable to test for such changes in channel trafficking in our experimental system. Alternatively, S70L might affect association with muscle-specific proteins or regulatory pathways that exist in skeletal muscle, but not in transfected HEK293T cells.

Mutations in *CLCN1* can lead to autosomal dominant or recessive myotonia congenita. In heterozygous patients a large fraction of hClC-1 channels consist of one WT and one mutant subunit, and the properties of such heterodimeric channels thus define the inheritance mode of myotonia caused by a given mutation. All patients of our study are heterozygous with two distinct *CLCN1* mutations on each allele, whereas relatives with only one mutation do not exhibit symptoms of myotonia congenita. We determined the surface membrane insertion of heterodimeric channels consisting of one WT and one Q43R subunit using two different methods. We first analyzed concatenated WT-Q43R dimers linked to a mYFP moiety. We constructed expression plasmids for both arrangements of the two coding regions, and these two constructs differed in apparent surface membrane insertion capability. Since concatenation might affect intracellular trafficking we determined surface membrane insertion of heterodimers that formed spontaneously in cells co-expressing WT and Q43R hClC-1. This analysis provided a reduction to 8% in surface insertion probability of heterodimeric channels. This value is closely similar to the results obtained for Q43R-WT, but not for WT-Q43R concatenated channels. Covalent linkage of two ClC-1 subunits potentially affects intracellular trafficking, possibly because some flexibility of the amino-terminus is necessary for normal intracellular trafficking.

The asymptomatic father of the index patient in family 1 carries the Q43R mutation only in one *CLCN1* allele and is thus expected to co-express WT and Q43R hClC-1 (Table 1). Our results on function, assembly and trafficking of hetero- and homodimeric Q43R hClC-1 channels predict - assuming equal expression of mutant and WT hClC-1 - a reduction of the sarcolemmal chloride conductance to about 25% in such heterozygous patients. This drastic reduction is expected to result in sarcolemmal hyperexcitability^10^,^12^,^49^,^50^ and is therefore in disagreement with the apparently recessive inheritance of this particular mutation. A possible explanation for recessive inheritance might be that the number of Q43R hClC-1 subunits is much lower than of WT subunits, either by decreased Q43R or increased WT expression^51^. In both cases, the percentage of heterodimeric channels would be lower than the value (50%) that is predicted for equal expression of WT and mutant subunits. Alternatively, myotonic symptoms might be modified by additional factors, some of which have already been identified^52^. The clinical symptoms of myotonia congenita are known to exhibit significant heterogeneity and variable expressivity^17^,^47^. One might thus imagine that the heterozygous father is not completely asymptomatic, but rather exhibit very mild symptoms that precluded diagnosis of myotonia congenita. Lastly, we cannot exclude that cultured cells and adult skeletal muscle might subtly differ in intracellular trafficking of homo- or heterodimeric channels.

So far, no functional roles have been assigned to within the amino-terminus or helices B and C of CLC proteins. Since all mutations reduce surface membrane insertion, one may conclude that the hClC-1 amino-terminus regulates intracellular trafficking, in agreement with recent findings on the role of amino-terminal retention signals in ClC-3^53^,^54^. Q43R causes the most pronounced trafficking effect in homodimeric mutant channels, and experiments with heterodimeric channels indicate that one mutant amino terminus is sufficient to retain hClC-1 channels in intracellular compartment.

In summary, we evaluated the functional consequences of four myotonia-associated *CLCN1* mutations that predict single amino acid substitutions in the amino-terminus of hClC-1. We found that none of these mutations affected ion permeation and gating properties of muscle chloride channels. However, three out of the four tested mutations impair surface membrane insertion of affected channels to variable extents. Knowledge about all functional alterations of hClC-1 channels will advance our understanding of myotonia congenita and is an important step towards rational therapeutic strategies.

## Methods

### Clinical data

The seven individuals included in this study were selected from patients who were referred to Dr. Sternberg’s laboratory in Hôpital Pitié-Salpêtrière for genetic testing after seeking medical advice for muscle stiffness and additional myotonic symptoms. The diagnosis of myotonia was based on clinical examination, with electromyography performed in four patients. Additional tests for increased creatine kinase activity or Steinert’s dystrophia myotonica protein kinase (DMPK) were performed. Clinical, electrophysiological and genetic data of the patients are summarized in Table 1. Family history was taken in patient interviews or from known case histories of parents and other relatives.

### Molecular genetic analysis

Mutation search in *CLCN1* and genetic diagnosis were performed according to and approved by French genetic diagnosis laws and according to the Declaration of Helsinki. An informed consent was given by each patient and recorded according to French law. The collection of patients‘ samples was declared and approved under number 646 by the Paris University hospitals. The diagnostic database was declared and approved under number 1412729 by the informatic databases French authority. Blood samples were taken after informed consent according to the French law and sent for genetic diagnosis of non-dystrophic myotonia. Genomic DNA was extracted from peripheral blood lymphocytes. The whole coding region of the *CLCN1* was amplified from genomic DNA by PCR, using 23 primer pairs (available upon request)^55^. Both forward and reverse sequence reactions were performed with the Big Dye Terminator Cycle Sequencing Ready Reaction Kit (Applied Biosystems, Foster City, CA). The sequence products were run on an ABI 3730 automated sequencer (Applied Biosystems), and data were analyzed with the Seqscape 2.1 software (Applied Biosystems).

The molecular genetics diagnosis for all patients was performed according to French law, after patients were informed about the nature and diagnostic aim of genetic tests and gave their consent to it.

### Expression of WT and mutant hClC-1 channels

The QuikChange site-directed mutagenesis kit (Stratagene, La Jolla, CA) was used to introduce point mutations into the plasmid pSVL-mYFP/CFP-hClC-1 containing the full length WT hClC-1 cDNA^31^ order of appearance with monomeric YFP or monomeric CFP covalently linked to the amino-terminus. To boost expression levels we subcloned mYFP-Q43R hClC-1 into the pRcCMV vector, and all electrophysiological (Figs 1 and 2) and combined fluorescence/electrophysiology data (Figs 3 and 4) provided in this manuscript were generated with cells transfected with pRcCMV-mYFP/CFP-Q43R hClC-1. In all cases, homodimeric channels were expressed by transfecting monomeric expression plasmids into HEK293T cells. To express a homogenous population of heterodimeric channels we constructed and transfected concatameric plasmids as described previously^44^,^56^. If not noted otherwise, mutant and WT hClC-1 channels were transiently expressed in HEK293T cells and examined typically two days after transfection of 0.2–2 μg cDNA with the calcium phosphate precipitation method^33^.

Our experimental approach required the use of fluorescent ClC-1 fusion proteins, and we decided to attach the mYFP moiety to the amino-terminus^38^. The resulting mYFP-hClC-1 has been intensely studied in the past, without any discernable differences in ion conduction, gating or regulation as compared to untagged hClC-1 channels. However, since sequence alterations in the amino-terminus have been reported to affect the subcellular localization of CLC channels and transporters^53^,^57^, this maneuver might affect intracellular trafficking of WT and mutant hClC-1. Identical whole-cell current amplitudes of HEK293T cells expressing hClC-1 (peak current amplitude at −135 mV: −7.55 ± 1.0 nA, n = 18 ) or mYFP-hClC-1 (peak current amplitude at −135 mV: −7.66 ± 0.79 nA, n = 27) indicate that linkage of mYFP to the hClC-1 amino-terminus does not cause major alterations of trafficking.

### Electrophysiology

Standard whole-cell patch clamp recordings were performed using an Axopatch 200B amplifier (Molecular Devices, Sunnyvale, CA USA). Pipettes were pulled from borosilicate glass and had resistances of 1.2–2.2 MΩ. More than 80% of the series resistance was compensated by an analog procedure, and the calculated voltage error due to series resistance was always <5 mV. Currents were filtered with an internal 4-pole Bessel filter with 1 kHz (-3dB) and digitized with sampling rates of 10–20 kHz using a Digidata AD/DA converter (Molecular Devices, Sunnyvale, CA USA). Cells were clamped to 0 mV for at least 2 s between two subsequent test sweeps. The standard internal solution contained (in mM): 120 NaCl, 2 MgCl_2_, 5 EGTA, 10 HEPES, pH 7.4; and the extracellular solution (in mM): 140 NaCl, 4 KCl, 2 CaCl_2_, 1 MgCl_2_, 5 HEPES, pH 7.4. For kinetic analyses, only current amplitudes below 10 nA were used, whereas for the determination of peak amplitudes cells with current amplitudes larger than 18 nA were omitted.

Data were analyzed by a combination of Clampfit (Molecular Devices, Sunnyvale, CA USA) and SigmaPlot (Systat Software Inc., San Jose, CA, USA) programs. Instantaneous current amplitudes were measured immediately after capacitive current relaxation (<500 μs after the voltage step). To obtain the relative probabilities of the channel to be open instantaneous current amplitudes after stepping to –125 mV were plotted versus the voltage of a preceding 300 ms pulse and normalized to the maximum tail current amplitude. The relative open probability of the slow gate was determined by measuring normalized tail current amplitudes after 300 ms prepulses to various potentials and a short fixed pulse of 0.5 ms to +180 mV, inserted prior to the test step^34^ (Fig. 2c). Activation curves were fit with a modified Boltzmann equation exhibiting a voltage-independent minimum open probability (P_min_) and a voltage-dependent term: P(V) = (P_max _− P_min_)/(1 + e^zδF(V −V0.5)/RT^) + P_min_, with zδ being the apparent gating charge, P_max_ the maximum open probability and V_0.5_ the midpoint of activation. All values are given as mean ± SEM.

### Noise analysis

Non-stationary noise-analysis was performed as previously described^31^. Currents were filtered at 10 kHz and digitized with a sampling rate of 50 kHz. A series of 50–300 sweeps was recorded upon voltage steps to −155 mV and pairs of subsequent records were then subtracted using the *Analysis* software (kindly provided by Dr. F. Bezanilla, University of Chicago, USA) to compute non-stationary ensemble variances^58^. Current variances were corrected for the background variance measured at the reversal potential (−5 mV) and sorted into evenly spaced current bins, with statistical errors superimposed as error bars.

hClC-1 channels exhibit a unique double-barreled architecture with two identical ion conduction pathways (protopores). For such channels with individual and common gating processes^33^,^59^ the current variance *σ*^2^ depends on the mean macroscopic current *I*, the number of active dimeric channels in the surface membrane *N* and the constant protopore current amplitude *i*, and also on the time-dependent open probabilities of protopore (*Pp(t*)) and common (*Pc(t*)) gates:


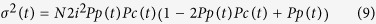


The macroscopic current is represented by


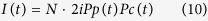


Thus, equation (1) can be converted into


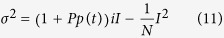


The single protopore current amplitude *i* can therefore be calculated by dividing the slope of the variance-current plot at low current amplitudes by the sum of one and the minimum absolute open probability of the protopore gate (1+Pp_min_)^33^.

### Simultaneous fluorescence and current recordings

For experiments correlating currents and the number of hClC-1-YFP fusion proteins, cells were cultivated on collagen coated glass cover slips and mounted in a perfusion chamber on an inverted IX71 microscope with UPlanSApo 60X/1.35 oil immersion objective (Olympus, Hamburg, Germany). mYFP was excited at 505 nm using a Polychrome V fast switching monochromator and emitted fluorescence light was detected at 530 nm using a PMT equipped ViewFinder III (Till Photonics, Gräfelfing, Germany)^42^,^60^. CFP was excited at 440 nm and detected at 470 nm. Fluorescence values were measured in the linear range of the photomultiplier and are given as arbitrary units (a.u.). Background fluorescence values and current amplitudes were measured on untransfected cells and found to be negligible. For multiple cells instantaneous current amplitudes at −135 mV were plotted versus fluorescence values, and these relationships fitted with linear functions. The slope factor correlates with the fraction of membrane inserted channels and is therefore a valuable parameter to quantify the mean membrane insertion efficiency ± the standard error of the fit.

In experiments on cells co-expressing WT hClC-1-YFP and Q43R hClC-1-CFP relative fluorescence values for YFP and CFP were used to determine relative expression of WT and mutant subunits. To account for different fluorescence amplitudes of individual mYFP or mCFP proteins, we determined the relative brightness factor (YFP/CFP = 3.0) of our experimental arrangement by comparing slopes of current-fluorescence plots for cells expressing WT hClC-1-YFP or WT hClC-1-CFP and corrected CFP fluorescence values by this factor.

### Confocal imaging

Live cell confocal imaging was performed on transiently transfected MDCK II cells grown on special confocal tissue culture treated dishes (Ibidi, Martinsried, Germany). Confocal images were obtained using a Leica DM IRB confocal microscope with a TCS SP2 AOBS scan head (Leica Microsystems, Wetzlar, Germany) after 2 days expression of fluorescent YFP or CFP fusion proteins of WT or mutant hClC-1. Protein biosynthesis was interrupted 10–20 min before imaging by cycloheximide (0.04 mg/ml) to reduce the amount of immature hClC-1 proteins localized in intracellular compartments.

### Biochemistry

To determine the expression levels of WT and mutant hClC-1-YFP fusion proteins (Fig. 1d) transfected HEK293T cells were lysed by incubation in 1% Triton X-100 in the presence of protease inhibitors (Sigma-Aldrich, Hamburg, Germany). After denaturation at room temperature in SDS sample buffer containing 100 mM dithiothreitol (DTT) cleared lysates were electrophoresed in parallel with fluorescent mass markers (Dual Color, Bio-Rad) on 10% SDS polyacrylamide gels. YFP-tagged proteins were visualized by scanning the wet PAGE gels with a fluorescence scanner (Typhoon, GE Healthcare, München, Germany), and individual bands were quantified with the ImageQuant software.To account for potential differences in cell numbers, the total protein amount of each lysate was determined using the BCA Protein Assay (Thermo Scientific). For each gel, lysate samples were diluted to obtain identical total protein amounts in all lanes containing WT and mutant hClC-1.

We studied plasma membrane expression and oligomeric assembly of WT and mutant hClC-1 upon heterologous expression in *Xenopus laevis* oocytes. WT and Q43R hClC-1 were fused in frame by sequences encoding an amino-terminal hexahistidine tag and a carboxy-terminal StrepII-tag (NWSHPQFEK)^61^ or with sequences encoding mYFP and subcloned into pGEMHE^61^. Capped cRNA was synthesized through use of MESSAGE machine kits (Ambion, Austin, TX, USA), and collagenase-treated, defolliculated stage IV–V oocytes were microinjected with 10 ng of RNA (and incubated at 18 °C in ND96 (in mM: 96 NaCl, 2 KCl, 1.8 CaCl_2_, 1 MgCl_2_, 5 Hepes, pH 7.6, supplemented with 2.5 sodium pyruvate and 50 μg/ml gentamycine sulfate). Proteins were metabolically labeled by incubating oocytes with ^35^S-methionine and cell surface-specific labeled using a membrane-impermeant Cy5-NHS ester. This system permits simple visual assessment of the intactness of individual oocytes using a stereo microscope and thus bypasses contamination of surface hClC-1 with intracellular channels labeled in leaky cells. hClC-1 channels were extracted by the mild non-ionic detergent digitonin as previously described^46^,^63^, purified via affinity chromatography using Ni-NTA-agarose (Qiagen, Hilden, Germany) and, in parallel, by Strep-Tactin^TM^ Sepharose (IBA, Göttingen, Germany) and resolved in the non-denatured state or SDS-denatured, dithiothreitol (DTT)-reduced state by blue native PAGE (BN-PAGE) and SDS-PAGE, as previously described^38^,^50^. PAGE gels were scanned first by a Typhoon fluorescence scanner and consecutively with a Phosphorimager to visualize the proteins through their fluorescence and incorporated ^35^S-methionine, respectively. Figures with images of PAGE gels were prepared with Image-Quant TL software version 7.0 (GE Healthcare Biosciences) for contrast adjustments, Adobe Photoshop CS2 for level adjustment, spot (dust/hot pixel) removal and cropping and Microsoft PowerPoint for labeling.

## Additional Information

**How to cite this article**: Ronstedt, K. *et al.* Impaired surface membrane insertion of homo- and heterodimeric human muscle chloride channels carrying amino-terminal myotonia-causing mutations. *Sci. Rep.* **5**, 15382; doi: 10.1038/srep15382 (2015).

## Supplementary Material

Supplementary Information

## Figures and Tables

**Table 1:** Clinical and molecular data of the investigated patients.

Family Nr	Familymember	Sex	Age	Symptomatic and/or anamnestic data	Electrophysiology data	Other data	Molecular data
366	index case	F	41	muscle stiffness that is alleviated by exercise	nd		c.128 A >G p.Gln43Arg (**Q43R**, exon 1) c.180 +3 A >T (intron 1)
	father	m	—	asymptomatic	nd		c.128 A >G p.Gln43Arg (Q43R, exon 1)
	mother	f	—	asymptomatic	nd		c.180 +3 A >T (intron 1)
427	index case	M	22	age at onset 15, severe muscle stiffness with moderate myalgia upon movement onset, ameliorated during continuation of effort, cold-insensitive	myotonic bursts, abundant (left short abductor pollicis, left biceps) or rare (left quadriceps), repeated short effort test: type III response	[CK] 424 UI absence of DMPK expansion	c.209 C >T p.Ser70Leu (**S70L**, exon 2) c.1471 +1 G >A (intron 13)
	father	m	—	asymptomatic	nd		c.209 C >T p.Ser70Leu (exon 2)
	mother	f	—	asymptomatic	nd		c.1471 +1 G >A (intron 13)
234	index case	M	29	muscle stiffness that is alleviated by exercise	nd		c.409 T >G P.Tyr137Asp (**Y137D**, exon 3) c.180 +3 A >T (intron 1)
	father	m	—	asymptomatic	nd		nd
	mother	f	—	asymptomatic	nd		nd
457	index case	M	37	myotonia with marked amelioration during exercise	nd		c.409 T >G P.Tyr137Asp (**Y137D**, exon 3) c.1488 G >T p.Arg496Ser (R496S, exon 14)
	affected sibling	M	—	symptoms identical to index case	nd		nd
	father	m	—	reported as affected on genetic tree, however no precise data available	nd		c.409 T >G P.Tyr137Asp (Y137D, exon 3) No other point mutation (all *CLCN1* and *SCN4A* exonic regions sequenced)
	mother	f	—	asymptomatic	nd		nd
1565	index case	F	32	myotonia	myotonic bursts	absence of DMPK expansion	c.409 T >G P.Tyr137Asp (**Y137D**, exon 3) c.2365—2 A >G (intron 19)
	father	m	—	nd	nd		nd
	mother	f	—	nd	nd		nd
1581	index case	M	35	age at onset 18, stiffness and myalgia the morning at awakening with correction by warm—up, muscle hypertrophy	myotonic bursts, abundant	[CK] 400–530 UI	c.409 T >G P.Tyr137Asp (**Y137D**, exon 3) c.2564 G >A p.Gly855Glu (G855E, exon 22)
	father	m	—	asymptomatic	nd		nd
	mother	f	—	asymptomatic	nd		nd
1541	index case	M	57	feeling of muscle stiffness needing a warm-up, muscle hypertrophy	myotonic bursts		c.480 G >C p.Gln160His (**Q160H**, exon 4) c.2680 C >T p.Arg894* (R894X, exon 23)
	father	m	—	nd	nd		nd
	mother	f	—	asymptomatic	nd		nd
