# Supplementary material for: Impaired surface membrane insertion of homo- and heterodimeric human muscle chloride channels carrying amino-terminal myotonia-causing mutations
Source: Sci Rep. 2015 Oct 27;5:15382. doi: 10.1038/srep15382 (PMC4621517; doi:10.1038/srep15382)
Supplement: Supplementary Information [file srep15382-s1.pdf]

Supplementary Information

Impaired surface membrane insertion of homo- and heterodimeric human muscle chloride channels carrying amino-terminal myotonia-causing mutations

Katharina Ronstedt[1], Damien Sternberg[2], Silvia Detro-Dassen[3], Thomas Gramkow[1], Birgit Begemann[1], Toni Becher[1], Petra Kilian[1], Matthias Grieschat[1], Jan-Philipp Machtens[4], Günther Schmalzing[3], Martin Fischer[1], and Christoph Fahlke[4]

[1]Institut für Neurophysiologie, Medizinische Hochschule Hannover, [2]Hôpital Pitié-Salpêtrière, Paris, [3]Abteilung Molekulare Pharmakologie, RWTH Aachen University, and [4]Institute of Complex Systems-Zelluläre Biophysik (ICS-4), Forschungszentrum Jülich, Germany

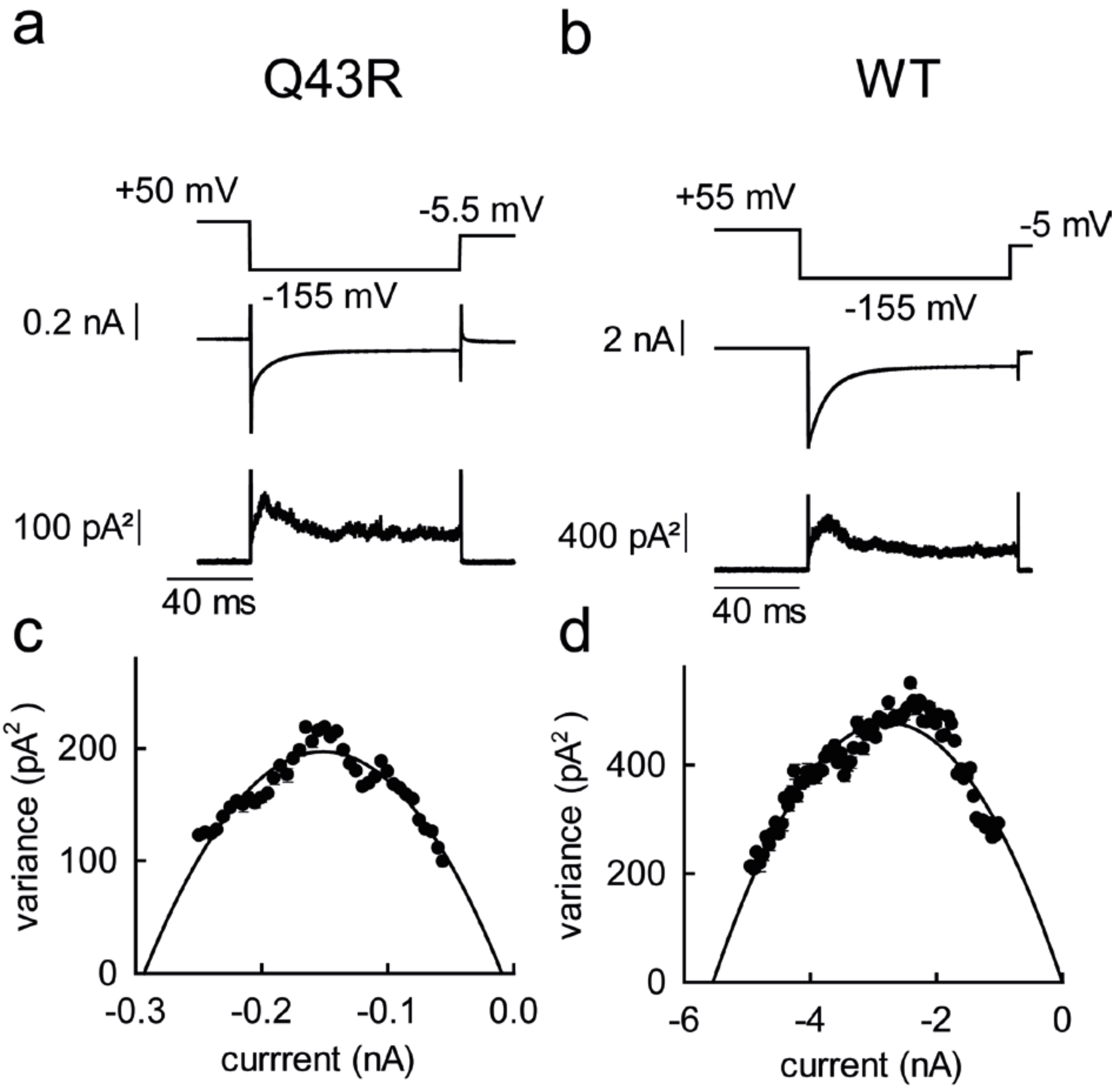

Figure S1 │ Myotonia-associated mutations do not affect ion conduction and absolute open probability of hClC-1. (a,c) Mean current responses and current variances for HEK293T cells expressing Q43R (a) or WT (c) hClC-1. Cells were transfected with 0.5 µg pSVL-mYFP-WT hClC-1 or 2 µg pRcCMV-mYFP/CFP-Q43R hClC-1. (b, d) Variance vs. current plots from the cells shown in (a) and (c). Solid lines represent quadratic functions fitted to the experimental data**.**

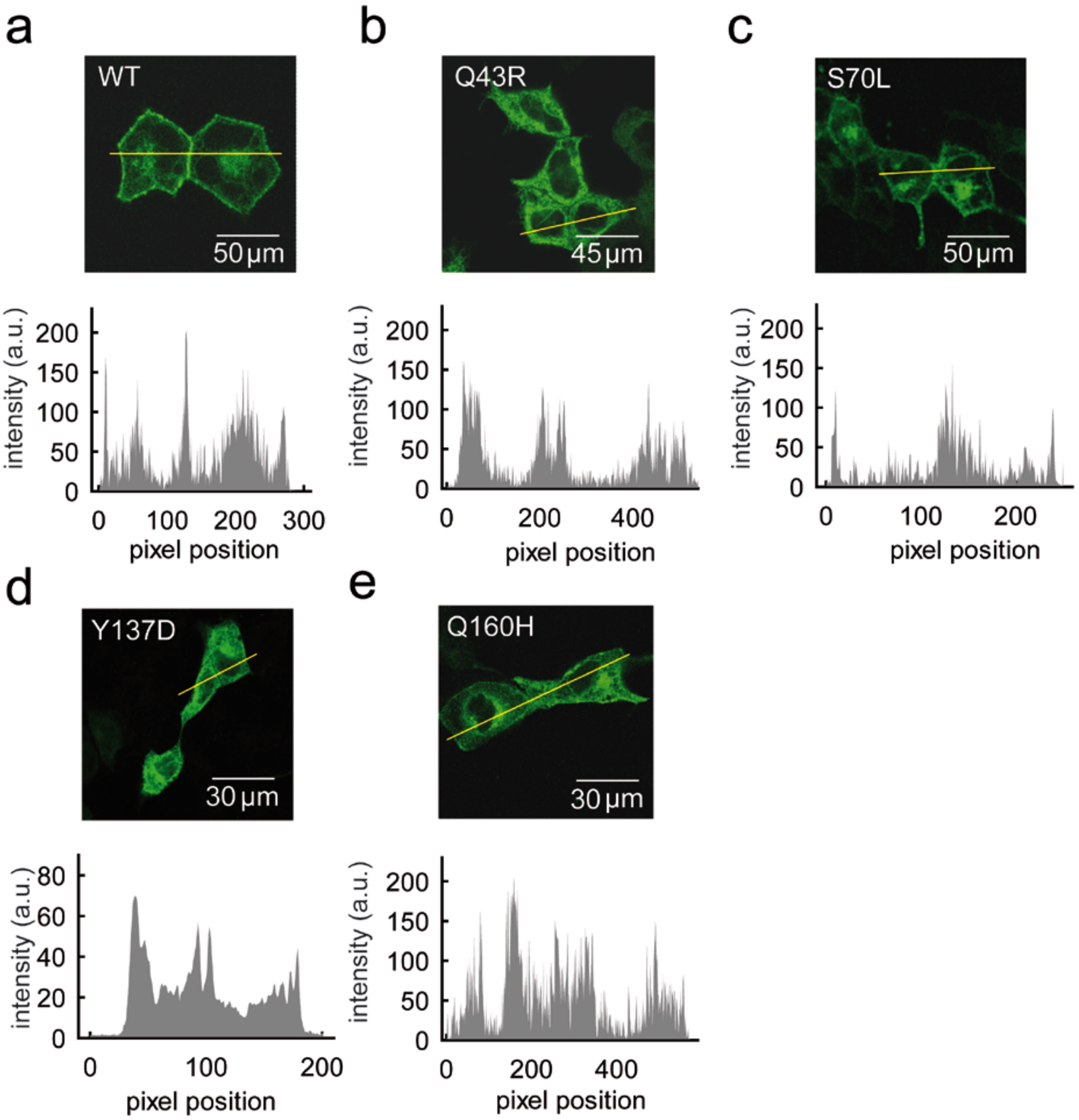

Figure S2 │ Representative confocal images from MDCK II cells expressing WT or mutant hClC-1-YFP. In these experiments, we transfected 0.2 µg pSVL-mYFP-hClC-1 for WT, 0.3-0.5 µg pSVL-mYFP-hClC-1 for S70L, Y137D, or Q160H hClC-1, or 0.5 µg pSVL -mYFP-Q43R hClC-1 for Q43R and incubated transfected cells for 48 hours.

a

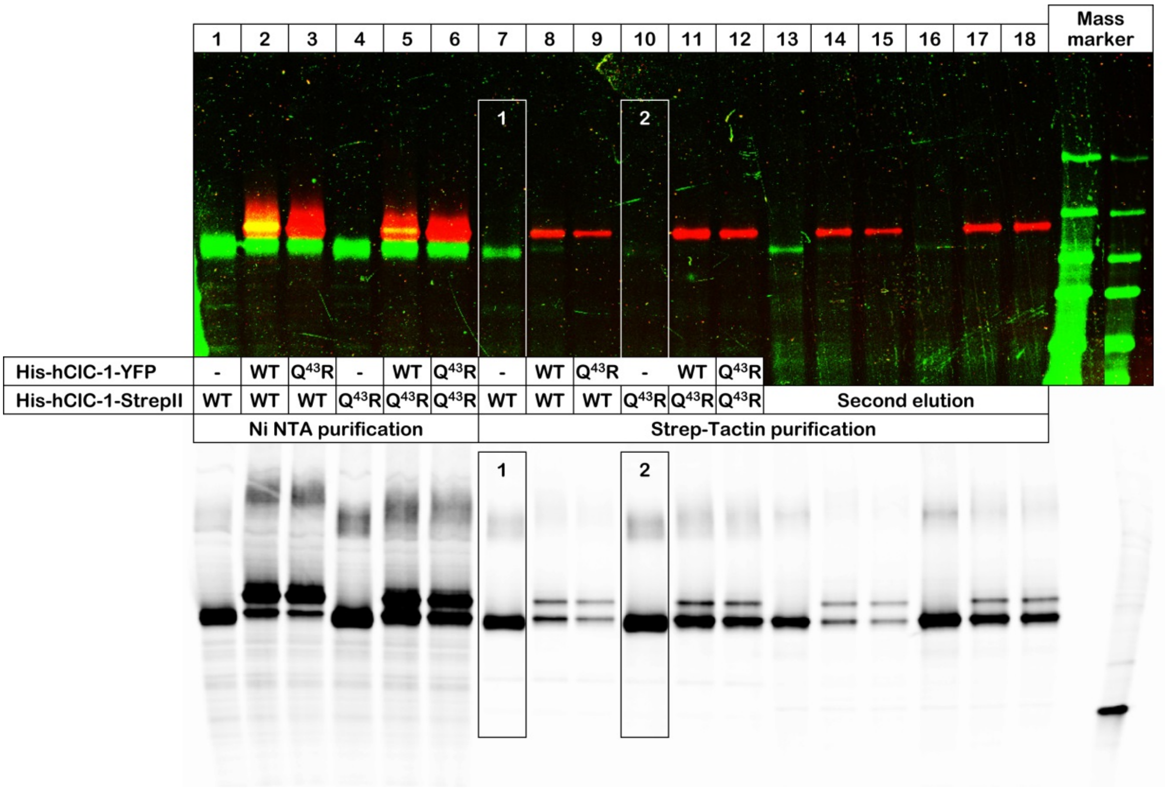

b

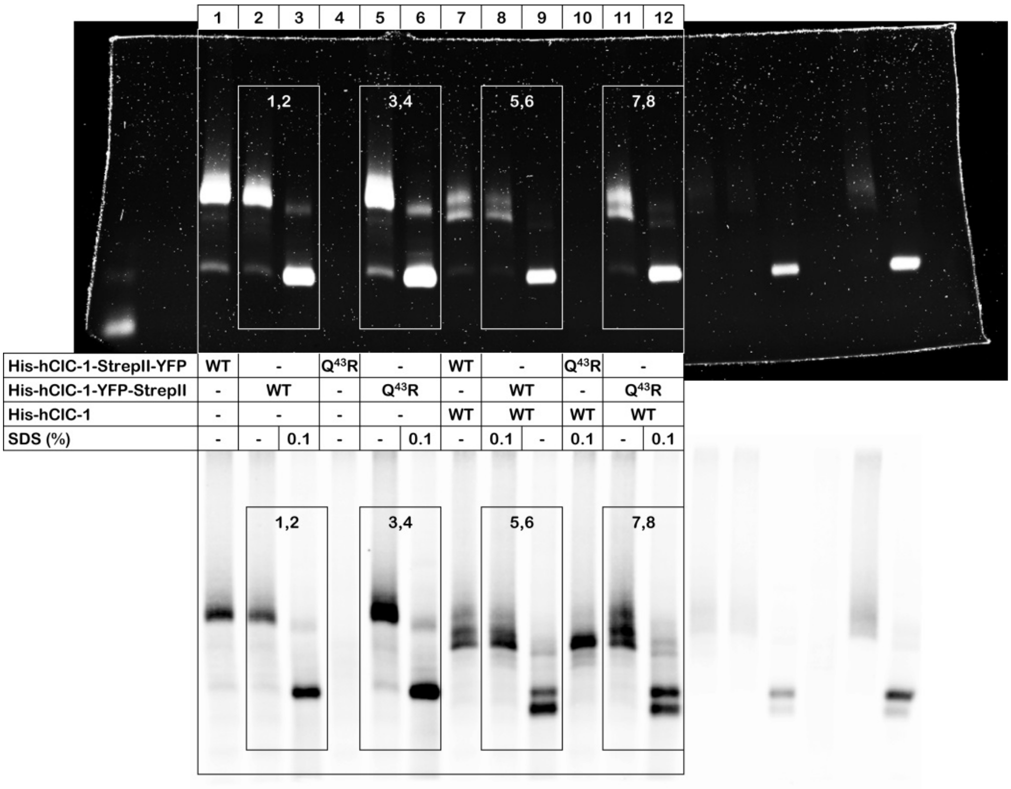

Figure S3 │ Source images of the cropped PAGE gel images shown in Fig. 5. (a) Source image of the cropped SDS PAGE gel in Fig. 5a. (b) Source image of the cropped BN PAGE

gel in Fig. 5b. For both graphs, Typhoon fluorescence scans are given in the upper panels and [^{35}S] phosphorimager scan lower panels. Each individual image was generated with the same ImageQuant settings adjusted to show relevant details. Rectangle lines indicate the lanes that were cropped out from the source image to generate Fig. 5a and b. The numbers within the rectangles refer to the gel lanes in Fig. 5a and b.
